# Supplementary material for: Elastic Bioresorbable Polymeric Capsules for Osmosis-Driven Delayed Burst Delivery of Vaccines
Source: Pharmaceutics. 2021 Mar 23;13(3):434. doi: 10.3390/pharmaceutics13030434 (PMC8004877; doi:10.3390/pharmaceutics13030434)
Supplement: Supplementary file 1 [file pharmaceutics-13-00434-s001.pdf]

pharmaceutics MDPI

Supplementary Materials: Elastic Bioresorbable Polymeric Capsules for Osmosis-Driven Delayed Burst Delivery of Vaccines

Kerr D.G. Samson, Eleonore C.L. Bolle, Mariah Sarwat, Tim R. Dargaville and Ferry P.W. Melchels

1. NMR Spectra Confirming Full Methacrylation

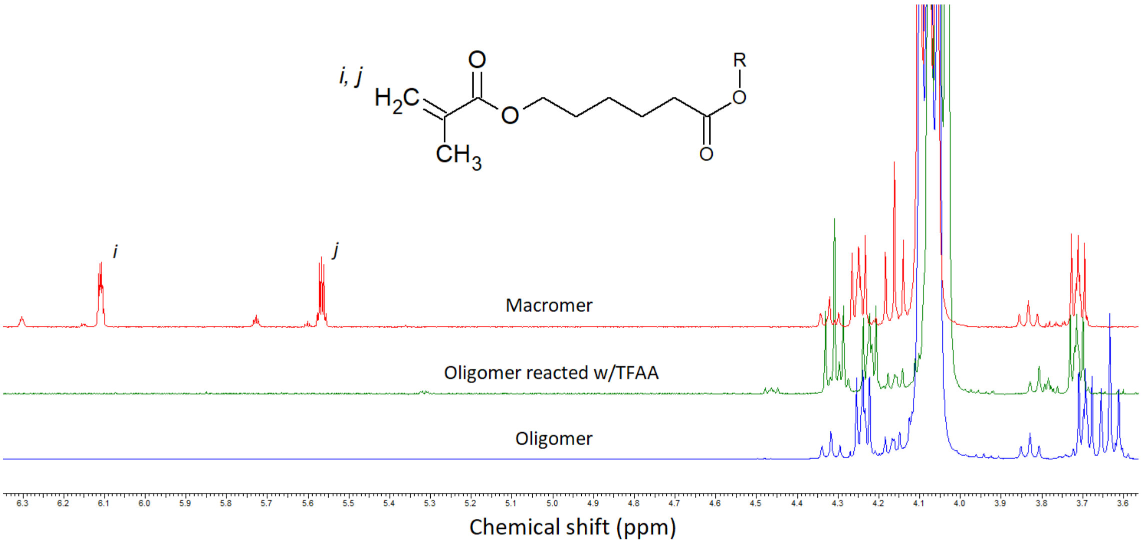

Figure S1. ^{1}H-NMR spectra of the 10 kg/mol oligomer (blue), TFAA reacted oligomer (green), and macromer (red). The peak centred at 3.65 ppm in the oligomer trace, which is associated with the penultimate methylene groups on the PCL end units, is shifted to approximately 4.3 ppm in the TFAA spectra or 4.15 ppm in the macromer spectra, respectively, as the reaction progresses. The macromer spectra shows the methacrylate end-group vinyl carbons at 5.57 and 6.11 ppm. Here, R represents the rest of the molecule. Traces offset for clarity. Impurity in the form of methacrylic anhydride reagent is present in the final product at 0.1% wt.

2. Basic Shorthand Structures of Thermoplastic Polymer and Crosslinkable Macromers

a) Thermoplastic PCL

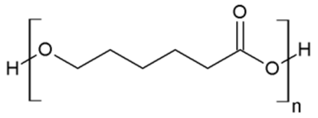

b) xPCL macromer (10,136 g/mol M_n)

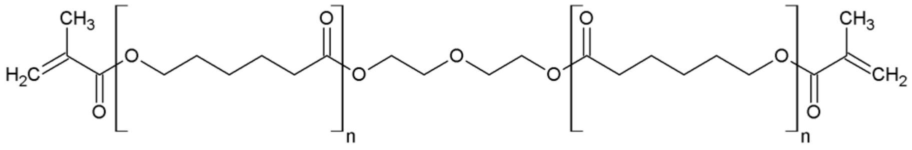

c) xPCL macromer (1,104 g/mol M_n)

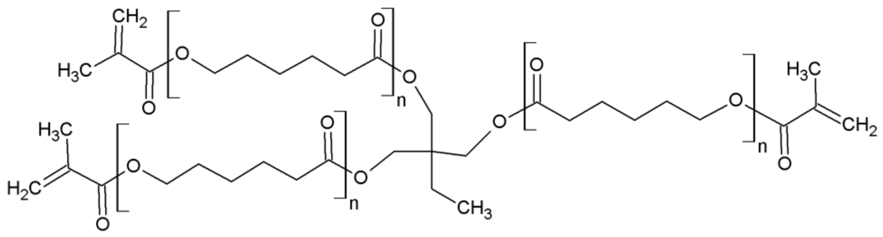

Figure S2. Basic shorthand structural formulae of (a) thermoplastic PCL, (b) linear bifunctional xPCL macromer unit, and c) three-armed trifunctional xPCL macromer unit. The xPCL material is composed of a mixture of b) and c). The 10,136 g/mol macromer b) is synthesised from the 10,000 g/mol oligomer, while the 1,104 g/mol macromer c) was synthesised from the 900 g/mol oligomer. During photo-curing, the macromers form covalent bonds with one another though their methacrylate end-groups. The thermoplastic PCL is not photo-curable, and instead polymer chains associate with each other through intermolecular forces.

3. Bulk Mechanical Properties

Table S1. Scaled down (-30% all dimensions) ASTM D638 type IV dumbbells were produced by injection moulding and photocuring (xPCL) or melt compression moulding (PCL). Tensile testing was performed at room temperature and humidity on a Instron 5567 chassis fitted with a 500 N load cell and side-action grips. An extension rate of 5 mm/min was used. Initial grip separation was 30 mm. Samples were extended until break.

Material	E (MPa)	Yield		Break	
		Stress (MPa)	Strain (%)	Stress (MPa)	Strain (%)
PCL	475.3 ± 15.0	21.6 ± 0.7	9.97 ± 0.8	31.36 ± 2.6	753.7 ± 103.3
xPCL	140.9 ± 14.2	8.7 ± 1.0	6.17 ± 0.6	12.6 ± 1.5	143.9 ± 19.0

4. Tubular part wall thickness determined by micro-computed tomography

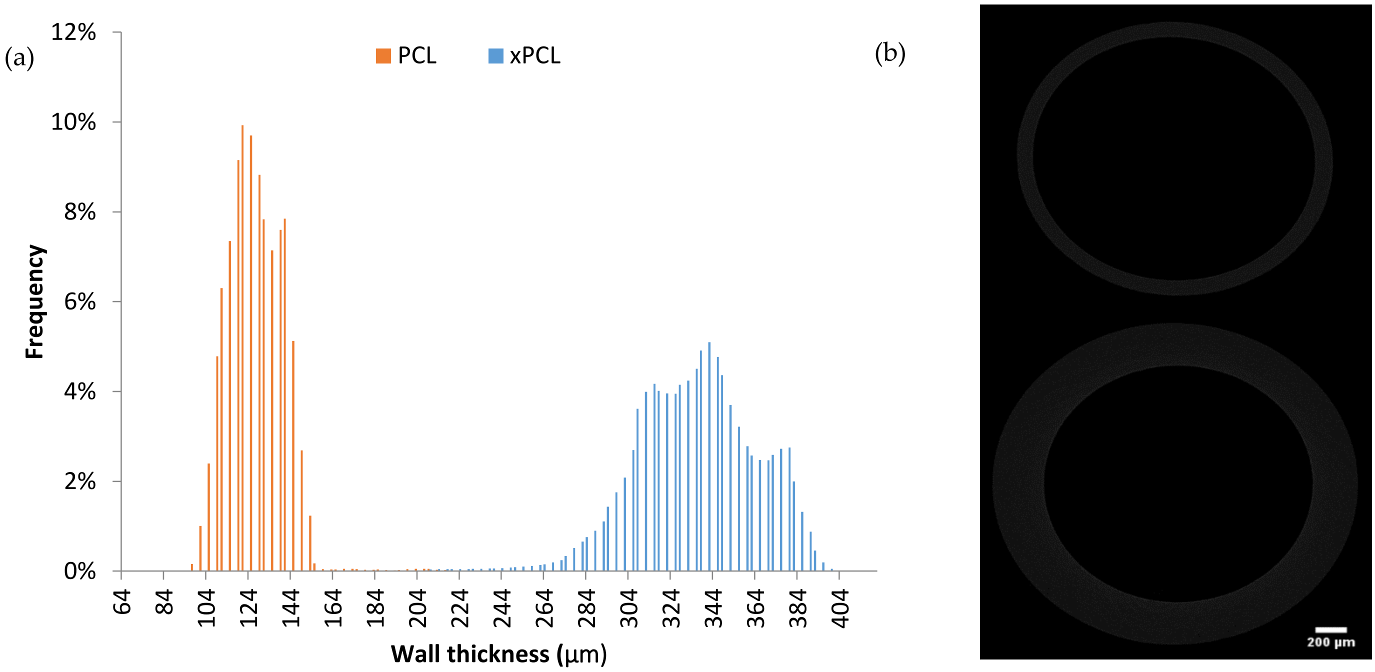

Figure S3. (a) Wall thicknesses displayed as histograms produced from scanning a PCL, and a xPCL tube. Bin size is in 2 µm increments. The number of counts within each bin is expressed as a percentage of the total number of counts. Both groups feature 2,541,600 measurements each determined from a single tube. Measurements were taken from the microCT images using an ImageJ macro. **(b)** Examples of microCT images/slices of a PCL tube (top) and xPCL tube (bottom). Scale bar indicates 200 µm.

5. Water Uptake Rate, Peak Mass, and Burst Delay Time for Individual Samples

Table S2. Average water uptake rate, peak mass, and average burst delay times of each device subgroup from each detection methodology, and their associated standard deviations (n = 3–5). There were 3 defective devices (PCL 3 m tube 3, PCL 3.5 m tube 4, and xPCL 3.5 m tube 4) determined by an immediate loss of dye. These defective devices were excluded from further data analysis (such as sub-group average calculations) and are highlighted below. The peak mass gain was determined from the highest mass value returned during the gravimetric measurements throughout the experiment. In each case, this was the last data point taken prior to burst, being directly followed by loss of mass.

Device Material	Osmolality (m)	Tube Number / Average	Water Uptake Rate (μg/day)	Peak Mass (mg)	Burst Delay Time (days)		
					Visual Detection	Gravimetric Monitoring	UV Spectro-Photometry
PCL	3	1	364.83	18.2	58	51	54
		2	418.867	15	44	42	44
		3	n/a	0.2	0	0	0
		4	411.11	11.1	37	37	35
		5	471.51	17.6	42	42	42
		**AVG ± SD**	**507 ± 45**	**15.5 ± 2.8**	**45.3 ± 7.8**	**43.0 ± 5.0**	**43.8 ± 6.8**
	3.5	1	669.03	12.4	26	21	21
		2	682.35	19.3	37	37	35
		3	990.9	20.1	21	21	21
		4	n/a	6.5	0	0	0
		5	657.74	13.5	26	23	23
		**AVG ± SD**	**770 ± 134**	**16.3 ± 3.4**	**22.0 ± 12.2**	**25.5 ± 6.7**	**25.0 ± 5.8**
	4	1	822.5	11.8	16	19	16
		2	820.23	17.4	26	26	23
		3	882.51	18	26	23	23
		4	795	12.9	26	21	21
		5	780.48	16.6	26	26	26
		**AVG ± SD**	**830 ± 34**	**15.3 ± 2.5**	**24.0 ± 4.0**	**23.0 ± 2.8**	**23.3 ± 1.8**
xPCL	3	1	642.86	7.7	21	19	16
		2	174.73	1.3	12	12	12
		3	449.83	25.8	56	58	56
		**AVG ± SD**	**470 ± 170**	**11.6 ± 10.4**	**29.7 ± 19.0**	**29.7 ± 20.2**	**28.0 ± 19.9**
	3.5	1	826.14	9.4	16	16	16
		2	784.19	15.2	26	26	23
		3	614.97	16.7	40	33	40
		4	n/a	-5.4	0	0	0
		**AVG ± SD**	**762 ± 59**	**13.8 ± 3.1**	**27.3 ± 9.8**	**25.0 ± 7.0**	**26.3 ± 10.1**
	4	1	854.14	11.5	21	21	19
		2	940.78	14.5	21	21	21
		3	854.1	10.9	21	19	19
		4	761.74	14.8	26	26	26
		**AVG ± SD**	**893 ± 93**	**12.9 ± 1.7**	**22.3 ± 2.2**	**21.8 ± 2.6**	**21.3 ± 2.9**

6. Release characteristics

Table S3. Displays the dye release at burst, average mass loss rate, and dye release rate of each device group, with the average mass lost and dye released by 72 at the end of the experiment for each group. The rates of mass loss and dye release were calculated over a range of days starting when the majority of the sub-group were observed to have entered steady state release and ended at day 72. The total mass released was calculated by taking the peak mass prior to burst and subtracting the mass measured at day 72. The total dye released by day 72 was calculated from the summation of dye release from all data points after burst detection, compared to the amount of dye that was loaded into the capsule.

Device Material	Osmolality (m)	Tube Number /Average	Dye Release at Burst (%)	Mass Loss Rate (µg/day)	Dye Release Rate (%/day)	Mass Lost by Day 72 (mg)	Dye Released by Day 72 (%)
PCL	3	1	0.44	201.4	0.2	4.2	5.60
		2	3.50	49.0	0.14	4.3	9.98
		3	n/a	412.5	1.31	22	105.51
		4	0.59	0.43	0.14	3.8	8.75
		5	0.84	38.1	0.17	5.1	8.52
		**AVG ± SD**	**1.3 ± 1.3**	**72.0 ± 76.7**	**0.16 ± 0.03**	**4.4 ± 0.5**	**8.2 ± 1.6**
	3.5	1	1.31	41.7	0.19	4.8	12.82
		2	1.11	48.0	0.19	5.7	9.14
		3	0.74	60.8	0.21	8.3	15.00
		4	n/a	128.32	0.34	13.4	57.71
		5	0.84	35.8	0.20	6.7	13.24
		**AVG ± SD**	**1.0 ± 0.2**	**47.0 ± 9.3**	**0.20 ± 0.01**	**6.4 ± 1.3**	**12.6 ± 2.1**
	4	1	1.04	37.2	0.67	3.5	39.67
		2	0.56	114.6	0.23	8	13.37
		3	1.09	75.7	0.27	9.4	14.23
		4	0.71	44.4	0.17	4.7	10.26
		5	0.84	70.8	0.2	6.4	12.20
		**AVG ± SD**	**0.85 ± 0.2**	**68.5 ± 27.4**	**0.31 ± 0.19**	**6.6 ± 2.0**	**18 ± 10.9**
xPCL	3	1	6.5	1.6	0.59	4.5	36.31
		2	4.4	24.1	0.53	3.4	37.55
		3	21.5	37.7	0.76	14.9	31.61
		**AVG ± SD**	**10.8 ± 7.6**	**21.0 ± 14.9**	**0.63 ± 0.10**	**7.6 ± 5.2**	**35.2 ± 2.6**
	3.5	1	1.8	23.4	0.48	3.4	34.54
		2	11.6	95.9	0.42	6.9	40.36
		3	14.3	86.5	0.49	11.2	31.49
		4	n/a	626.1	0.12	27.2	66.03
		**AVG ± SD**	**9.2 ± 5.4**	**69.0 ± 32.2**	**0.47 ± 0.03**	**7.2 ± 3.2**	**35.5 ± 3.7**
	4	1	3.3	15.9	0.45	2.7	39.39
		2	8.2	74.3	0.40	14.4	39.71
		3	5.4	24.3	0.47	6.8	34.85
		4	9.1	10.0	0.60	9.7	38.47
		**AVG ± SD**	**6.5 ± 2.3**	**31.0 ± 25.4**	**0.48 ± 0.07**	**8.4 ± 4.3**	**38.1 ± 1.9**

7. Sample crystallinity - DSC

Material	T_m (°C)	ΔH_m (µV/mg)
PCL 45 kg/mol	67	280
xPCL network	57	167

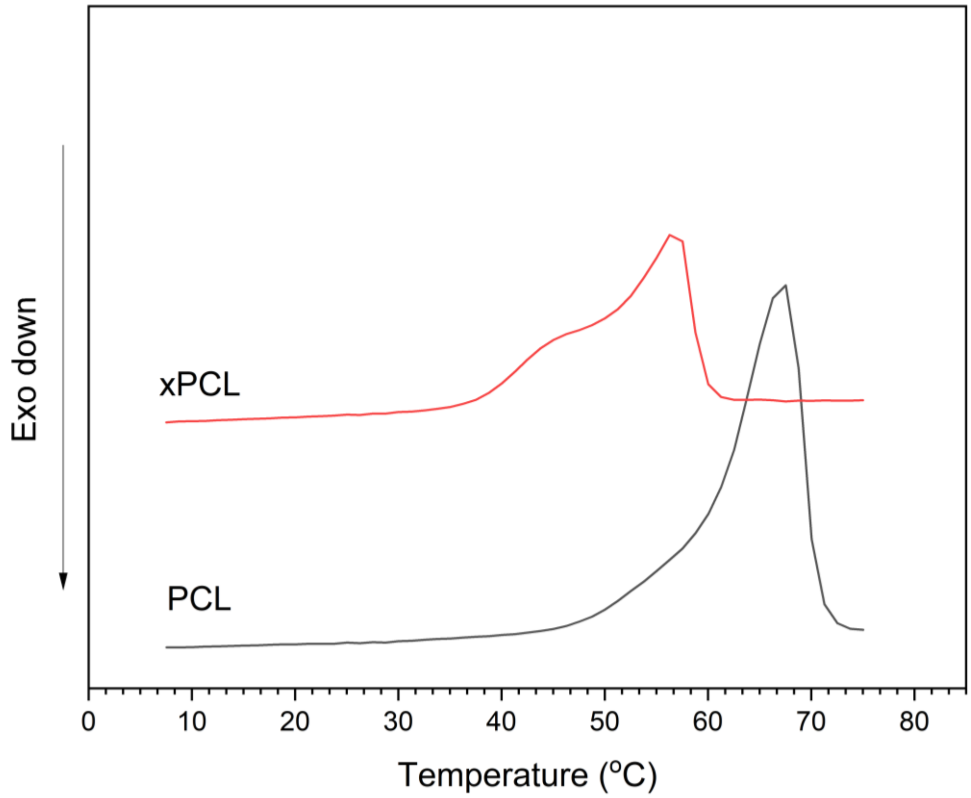

Figure S4. Compares the melting temperature (T_m) and melting enthalpy (ΔH_m) of the xPCL network to a commercially available thermoplastic PCL polymer (45 kg/mol) which is similar to the PCL material used to produce the PCL tubes in this study. Samples were stabilised at −20 °C before performing at heating ramp at 5 °C/min to 100 °C. The melting temperature was determined from the peak of the melting curve, while the melting enthalpy was determined from the integral of the melt peak. The xPCL network is composed of both 10,000 and 900 g/mol macromer units. The PCL material used to fabricate the tubes was of a higher molecular weight (70,000 g/mol). Traces are offset for clarity.
